# Supplementary material for: Simple growth conditions improve targeted gene deletion in Cryptococcus neoformans
Source: mSphere. 2025 Apr 2;10(4):e01070-24. doi: 10.1128/msphere.01070-24 (PMC12039239; doi:10.1128/msphere.01070-24)
Supplement: Table S1 — Primers. [file msphere.01070-24-s0001.pdf]

Supplemental Table 1_Primer Used In Study

Gene locus	Primer	Sequence (5' to 3')	Purpose	Product
CAS9	Cas9_P1	GTGTGGAATTGTGAGCGGAT	pair with Cas9_P2	6555bp
	Cas9_P2	GGTTTTCCCAGTCACGACGT	pair with Cas9_P1	
CNAG_02294_ ADE2 gRNA	gRNA_P1	TGTAAAACGACGGCCAGT	1st round of PCR for 5'arm (pair with gRNA_P2)	390bp
	gRNA_P2_ ADE2	tgttcctaacgaccataaccAACAG TATACCCTGCCGGTG	1st round of PCR for 5'arm (pair with gRNA_P1) Red sequence is overhang complement gRNA_P3	
	gRNA_P3_ ADE2	GGTTATGGTCGTTAGGAACAGTTTT AGAGCTAGAAATAGCAAGTT	1st round of PCR for 3'arm (pair with gRNA_P4) Red sequence is overhang complement gRNA_P2	233bp
	gRNA_P4	AGCGGATAACAATTTCACACAGG	1st round of PCR for 3'arm (pair with gRNA_P3)	
	gRNA_P5	CCATCGATTTGCATTAGAACTAAAA ACAAAGCA	2nd round of PCR for full fragment (pair with gRNA_P6)	399bp
	gRNA_P6	CCGCTCGAGTAAAACAAAAAAGCAC CGACTC	2nd round of PCR for full fragment (pair with gRNA_P5)	
CNAG_03465_ LAC1 gRNA	gRNA_P1	TGTAAAACGACGGCCAGT	1st round of PCR for 5'arm (pair with gRNA_P2)	390bp
	gRNA_P2_ LAC1	CCAAACATACAGCCCAGAGCCAACA GTATACCCTGCCGGTG	1st round of PCR for 5'arm (pair with gRNA_P1) Red sequence is overhang complement gRNA_P3	
	gRNA_P3_ LAC1	GCTCTGGGCTGTATGTTTGGGTTTT AGAGCTAGAAATAGCAAGTT	1st round of PCR for 3'arm (pair with gRNA_P4) Red sequence is overhang complement gRNA_P2	233bp
	gRNA_P4	AGCGGATAACAATTTCACACAGG	1st round of PCR for 3'arm (pair with gRNA_P3)	
	gRNA_P5	CCATCGATTTGCATTAGAACTAAAA ACAAAGCA	2nd round of PCR for full fragment (pair with gRNA_P6)	399bp
	gRNA_P6	CCGCTCGAGTAAAACAAAAAAGCAC CGACTC	2nd round of PCR for full fragment (pair with gRNA_P5)	
CNAG_02294_ ADE2 Deletion	ADE2_P1	CTCCGTGTACCACGCTGC	1st round of PCR for 5'arm (pair with ADE2_P4)	1358bp
	ADE2_P4	CTGGCCGTCGTTTTACCCACCG CCTGAGGATG	1st round of PCR for 5'arm (pair with ADE2_P4) Red sequence is overhang complement ADE2_P3	
	ADE2_P3	CATCCTCAGGCGGTGGGTAAAA CGACGGCCAG	1st round of PCR for Marker (pair with ADE2_P6) Red sequence is overhang complement ADE2_P4	1925bp
	ADE2_P6	CCTCCTCCAGAATGTCGACCAG GAAACAGCTATGAC	1st round of PCR for Marker (pair with ADE2_P3) Red sequence is overhang complement ADE2_P5	
	ADE2_P2	GTCATAGCTGTTTCCTGGTCGA CATTCTGGAGGAGG	1st round of PCR for 3'arm (pair with ADE2_P5)	1295bp
	ADE2_p5	GAAGATGAGGATATTGCCGAAG	1st round of PCR for 3'arm (pair with ADE2_P2) Red sequence is overhang complement ADE2_P6	
	ADE2_P7	GTGTCGATGGCAGATCTCC	2nd round of PCR for full fragment (pair with ADE2_P8)	3962bp

	ADE2_P8	GTCGGTCAAAGGACATCTG	2nd round of PCR for full fragment (pair with ADE2_P7)	3962bp
CNAG_02294_ ADE2 Deletion 50bp	ADE2_F_50	tcaataaatttctgcatcttcatctctctgacatccgcaccccttgtagcCTATGACCATGATTACGCCAAG	pair with ADE2_R_50	1901bp
	ADE2_R_50	cagttacagatatcgcaccaatctatgcatctgaccaacatagaaccgttCTGCGAGGATGTGAGCTG	pair with ADE2_F_50	
CNAG_03465_ LAC1 Deletion 50bp	LAC1_F_50	actctatgctaataggtctattcaatgtagctataccgacactcacagtaCTATGACCATGATTACGCCAAG	pair with LAC1_R_50	1901bp
	LAC1_R_50	tctcgtccaagtcgaagtgcccaataattcttcctcgtatcacttccaatCTGCGAGGATGTGAGCTG	pair with LAC1_F_50	
CNAG_02294_ ADE2 5' Screen	ADE2_check1	GAGGACAAATTACTCGGCATTC	PCR screen 5' (combine with ADE2_check2 and NAT_Check_5')	WT322bp KO 402bp
	ADE2_check2	CACCGCCTGAGGATGATTAG	PCR screen 5' (combine with ADE2_check1 and NAT_Check_5')	
	NAT_Check_5'	GAATCCGAGACAGACATCGTG	PCR screen 5' (combine with ADE2_check1 and ADE2_check2)	
CNAG_02294_ ADE2 3' Screen	ADE2_check3	CTTGAGGGTGGAAGCGAGAT	PCR screen 3' (combine with ADE2_check4 and NAT_Check_3')	WT386bp KO521bp
	ADE2_check4	CGCTTGAGGAAGAAGTTTTGG	PCR screen 3' (combine with ADE2_check3 and NAT_Check_3')	
	NAT_Check_3'	GGTGACGCTGTGAGAGTGGT	PCR screen 5' (combine with ADE2_check3 and ADE2_check4)	
CNAG_03465_ LAC1 5' Screen	LAC1_check1	cttccatgaaaggatggattgtc	PCR screen 5' (combine with LAC1_check2 and NAT_Check_5')	WT358bp KO559bp
	LAC1_check2	ggatcttgtgaagctcaacg	PCR screen 5' (combine with LAC1_check1 and NAT_Check_5')	
	NAT_Check_5'	GAATCCGAGACAGACATCGTG	PCR screen 5' (combine with LAC1_check1 and LAC1_check2)	
_03465_ LAC1 3' Screen	LAC1_check3	gatgagcatttattgatgaagtcc	PCR screen 3' (combine with LAC1_check4 and NAT_Check_3')	WT459bp KO361bp
	LAC1_check4	gctccgacattaacccattaaac	PCR screen 3' (combine with LAC_check3 and NAT_Check_3')	

CNAG 3	NAT_Check_3'	GGTGACGCTGTGAGAGTGGT	PCR screen 5'<br>(combine with LAC1_check3 and LAC1_check4)	
